# Racial Residential Segregation and Mental Health During Pregnancy

**DOI:** 10.1001/jamahealthforum.2024.3669

**Published:** 2024-10-25

**Authors:** Kendria Kelly-Taylor, Sylvia E. Badon, Wendy T. Dyer, Alex Asera, Huyun Dong, Tess Baker, Nerissa Nance, Kiarri N. Kershaw, Charles P. Quesenberry, Kelly C. Young-Wolff, Mibhali Bhalala, Kathryn Erickson-Ridout, Lyndsay A. Avalos

**Affiliations:** 1 Division of Research, Kaiser Permanente Northern California, Pleasanton; 2 School of Public Health, University of California Berkeley, Berkeley; 3 Department of Preventive Medicine, Northwestern University Feinberg School of Medicine, Chicago, Illinois; 4 Department of Psychiatry and Behavioral Sciences, University of California, San Franciso

## Abstract

**Importance:**

Research suggests the social, physical, and socioeconomic contexts of residing in segregated neighborhoods may negatively affect mental health.

**Objective:**

To assess the association between racial residential segregation and prenatal mental health among Asian, Black, Hispanic, and White individuals.

**Design, Setting, and Participants:**

This population-based cross-sectional study was conducted in Kaiser Permanente Northern California (KPNC), an integrated health care delivery system. Participants included self-identified Asian, Black, Hispanic, and White pregnant individuals who attended at least 1 prenatal care visit at KPNC between January 1, 2014, and December 31, 2019. Data were analyzed from January 14, 2023, to August 15, 2024.

**Exposures:**

Racial residential segregation, defined by the local Getis-Ord Gi* statistic, was calculated in each racial and ethnic group and categorized as low (<0), medium (0-1.96), or high (>1.96). A positive Gi* statistic indicates overrepresentation (greater clustering or segregation) of the racial and ethnic group in an index census tract and neighboring tracts compared with the larger surrounding geographic area.

**Main Outcomes and Measures:**

Prenatal depression and anxiety defined by diagnoses codes documented in the electronic health record between the first day of the last menstrual period and the day prior to birth.

**Results:**

Among the 201 115 participants included in the analysis (mean [SD] age, 30.8 [5.3] years; 26.8% Asian, 6.6% Black, 28.0% Hispanic, and 38.6% White), prenatal depression and anxiety were highest in Black individuals (18.3% and 18.4%, respectively), followed by White (16.0% and 18.2%, respectively), Hispanic (13.0% and 14.4%, respectively), and Asian (5.7% and 6.4%, respectively) individuals. Asian (40.8% vs 31.1%) and Black (43.3% vs 22.6%) individuals were more likely to live in neighborhoods with high vs low segregation, while Hispanic individuals were equally likely (34.3% vs 34.7%). High compared with low segregation was associated with greater odds of prenatal depression (adjusted odds ratio [AOR], 1.25 [95% CI, 1.10-1.42]) and anxiety (AOR, 1.14 [95% CI, 1.00-1.29]) among Black individuals. High segregation was associated with lower odds of prenatal depression among Asian (AOR, 0.75 [95% CI, 0.69-0.82]), Hispanic (AOR, 0.88 [95% CI, 0.82-0.94]), and White (AOR, 0.91 [95% CI, 0.86-0.96]) individuals. Similar associations were found for anxiety among Asian (AOR, 0.80 [95% CI, 0.73-0.87]) and Hispanic (AOR, 0.88 [95% CI, 0.82-0.93]) but not White (AOR, 0.95 [95% CI, 0.90-1.00]) individuals.

**Conclusions and Relevance:**

In this cross-sectional study, racial and ethnic residential segregation was associated with worse prenatal mental health for Black individuals but better mental health for Asian, Hispanic, and White individuals. Policies reducing segregation and its impact may improve mental health outcomes in pregnant Black individuals.

## Key Points

**Question:**

Is racial residential segregation associated with prenatal mental health conditions among different racial and ethnic groups?

**Findings:**

In this cross-sectional study of 201 115 pregnant individuals in Northern California, high racial residential segregation was associated with increased odds of prenatal depression and anxiety among Black pregnant individuals and lower odds of depression and anxiety among Asian, Hispanic, and White pregnant individuals.

**Meaning:**

These findings suggest that policies reducing segregation of Black neighborhoods may mitigate these negative effects among Black populations.

## Teaser

This cross-sectional study evaluates the association between racial and ethnic residential segregation and prenatal depression and anxiety among pregnant Asian, Black, Hispanic, and White individuals universally screened for mental health conditions during pregnancy.

## Introduction

Prenatal depression and anxiety are maternal mental health conditions with significant adverse multigenerational health consequences for mothers and children that disproportionately affect Black and Hispanic individuals.^1,2^ Historically, differences in socioeconomic (eg, income, educational attainment, and employment),^3,4^ psychological (eg, increased psychological distress and allostatic load),^5,6^ and neighborhood (eg, poverty and social cohesion)^4,7,8^ factors have been explored to explain greater experiences of mental health conditions among these populations. However, given the complexities of individual and neighborhood factors and their mechanisms,^9^ it is important to investigate structural determinants and their impact on the ongoing racial and ethnic disparities in mental health conditions.

A growing body of research supports racial residential segregation, a dimension of structural racism, as a fundamental cause of health disparities (specifically the disparity between Black and White individuals).^10^ Racial residential segregation has created and reinforced racial inequalities in health by limiting economic and educational opportunities among racial and ethnic minority communities due to limited investment in economic infrastructure and environmental sustainability.^10^ Racial residential segregation is the degree to which 2 or more racial groups live apart from each other,^11^ and it may adversely impact health by limiting access to good-quality social and built environments, creating food deserts, limiting walkability, increasing exposure to toxins and pollutants, limiting high-quality education and employment opportunities, restricting access to health care, and increasing psychological stress.^12^ In the US, this separation was enforced through institutional policies, including discriminatory housing and lending practices, restrictive zoning legislation, and federal housing programs overwhelmingly impacting Black communities.^13^ Previous studies suggest that racial and ethnic residential segregation (hereinafter termed *racial residential segregation*) could be the common driver of individual and neighborhood level factors related to mental health conditions, thus serving as a plausible mechanism that underpins ongoing racial and ethnic disparities in mental health conditions.^14,15,16,17^

Studies from nonpregnant populations^18,19,20^ suggest that racial residential segregation may represent an important contributor to adverse mental health, but findings are mixed and differ by race and ethnicity. For example, some studies have found that high racial residential segregation is associated with an increase in depression symptoms in Black adults^19^ and depression and anxiety symptoms in Hispanic adults.^20^ However, a buffering effect of high racial residential segregation on mental health outcomes among Asian^20^ and Hispanic^17^ populations has also been observed. It is possible that for some racial and ethnic minority groups, residing in neighborhoods with high concentration of individuals who identify as the same racial and ethnic group may foster strong social networks, reinforcing social control and shielding these groups from exposure to prejudice and discrimination based on race and ethnicity, thus buffering against poor mental health.^21^ Among Black populations, the salubrious effects of ethnic density on depression symptoms have been observed to operate up to a certain threshold, above which they are undermined by the negative effects of extreme racial residential segregation.^22^

Research is lacking on the impact of racial residential segregation on mental health during pregnancy and its role in racial and ethnic disparities in mental health in this vulnerable population. The only study to date in pregnancy^23^ had several limitations, including a small sample, exclusion of depression diagnoses, and a setting in a single city in the Southern US. However, the investigators found that Black and Hispanic pregnant individuals residing among economic and racially homogenous groups reported lower prenatal symptoms.^23^ Furthermore, literature on this topic among pregnant Asian populations is sparse. Better understanding how racial residential segregation impacts prenatal mental health could inform potential policy and clinical intervention efforts for improving the health of new mothers and their children. The objective of this study was to evaluate the association between racial residential segregation and prenatal mental health outcomes (depression and anxiety) among pregnant Asian, Black, Hispanic, and White individuals universally screened for mental health conditions during pregnancy in a 22-county region in Northern California.

## Methods

### Study Setting

This population-based cross-sectional study was conducted within Kaiser Permanente Northern California (KPNC), a large, integrated health care delivery system that provides care in 22 counties and serves a population of approximately 4.6 million members (66 000 pregnancies annually). KPNC health plan members are covered by employee-sponsored insurance plans, the California insurance exchange, Medicare, and Medicaid and are broadly representative of the local and statewide population.^24^ As part of standard prenatal care at KPNC, pregnant individuals are universally screened for depression and anxiety and, when appropriate, receive a clinical diagnosis that is recorded in the KPNC electronic health record (EHR) system.^25^ This study followed the Strengthening the Reporting of Observational Studies in Epidemiology (STROBE) guidelines for cross-sectional studies and was approved by the KPNC Institutional Review Board. The board waived the requirement of informed consent. Study procedures meet Health Insurance Portability and Accountability Act requirements and 42 CFR part 2 regarding medical records. Upon enrollment in the health plan, all KPNC members are informed that their data may be used for research.

### Study Population

We included all individuals with singleton pregnancies resulting in live births between January 1, 2014, and December 31, 2019, who were enrolled in the KPNC health plan during pregnancy, attended at least 1 prenatal care visit, and self-reported their racial and ethnic group as Asian, Hispanic, non-Hispanic Black (hereinafter referred to as Black), or non-Hispanic White (hereinafter referred to as White). We excluded American Indian or Alaska Native and Native Hawaiian or Other Pacific Islander groups due to the small sample sizes; we excluded multiracial pregnant individuals given the heterogeneity within this group.

### Exposure

Racial and ethnic residential segregation was evaluated separately for Asian, Black, Hispanic, and White individuals using the Getis-Ord Gi* statistic,^26,27^ which was calculated using the geocoded residential address at the beginning of pregnancy linked to census tract-level population estimates from the same year as the pregnancy.^28^ The Gi* statistic is a widely used measure of segregation in epidemiology.^29,30,31,32,33,34^ It measures clustering, 1 of the 5 dimensions of segregation defined by Massey and Denton, which quantifies the extent to which racially and ethnically similar neighborhoods group together in space.^11^ Each census tract receives a *z* score for each racial and ethnic group indicating the extent (number of SDs) to which the proportion of residents of that racial and ethnic group in the index census tract and neighboring tracts (tracts that share a border with the index tract) deviates from the mean proportion of residents of that racial and ethnic group in a larger surrounding geographic area (core-based statistical area for individuals living in metropolitan areas; county for individuals living in nonmetropolitan areas).

A positive Gi* statistic indicates overrepresentation (greater clustering or segregation) of the racial and ethnic group in the index census tract and neighboring tracts compared with the larger surrounding geographic area. Gi* statistics near zero indicate similar representation of the racial and ethnic group in the index census tract and neighboring tracts compared with the larger surrounding geographic area. A negative Gi* statistic indicates underrepresentation of the racial and ethnic group in the index census tract and neighboring tracts compared with the larger surrounding geographic area. Using a type I error rate of 5% to define statistical significance, a Gi* statistic of 1.96 corresponds to a statistically significant difference in racial and ethnic group representation in the index census tract and neighboring tracts compared with the larger surrounding geographic area.

Each individual was categorized into high (Gi* statistic, >1.96), medium (Gi* statistic, 0-1.96), and low (Gi* statistic, <0) residential segregation for their self-reported racial and ethnic group. These are standard cutoffs used in studies of racial residential segregation and health outcomes.^29,30,31,32,33,34^

### Outcomes

Prenatal depression and anxiety diagnoses were defined by *International Classification of Diseases, Ninth Revision, Clinical Modification* and the *International Statistical Classification of Diseases, Tenth Revision, Clinical Modification* codes documented in the EHR between the first day of the last menstrual period and the day prior to a live birth (see eTable 1 in Supplement 1 for the codes). Depression severity was defined by self-report responses to the Patient Health Questionnaire (PHQ-9) completed as part of standard prenatal care. The PHQ-9 is a validated instrument for screening for depression across racial and ethnic groups with high sensitivity (>88%) and specificity (>88%) among patients using obstetrical services.^35,36^ Depression severity was categorized by PHQ-9 score as none or mild (0-9), moderate (10-14), or severe (15-27).

### Study Covariates

Study covariates ascertained from the EHR database included maternal age, partnered status, parity, self-reported substance use during pregnancy at entry into prenatal care (smoking, alcohol, and other substances), educational attainment, insurance payer, and neighborhood deprivation. Neighborhood deprivation was defined by quartile of the neighborhood deprivation index (NDI).^37^

### Statistical Analysis

Data were analyzed from January 14, 2023, to August 15, 2024. All analyses were stratified by racial and ethnic group. Descriptive statistics were summarized by racial residential segregation category to describe the study sample. Multilevel logistic regression models were used to estimate association of racial residential segregation category with each maternal mental health outcome (depression diagnosis, moderate depression, severe depression, or anxiety diagnosis), adjusting for a priori identified confounders (ie, maternal age in years, partnered status [partnered, not partnered, other, or unknown], smoking [yes or no], alcohol use [yes or no], and substance use [yes or no] in early pregnancy). Models included random intercepts for census tract, to account for clustering within census tracts, and for participant, to account for nonindependence of multiple pregnancies in the same participant. No or mild depression was the reference category for models with moderate or severe depression as outcomes.

We conducted 2 sensitivity analyses. The first analysis limited the sample to pregnant individuals in urban areas (there were too few residents of rural areas in our study to stratify) because mechanisms through which racial residential segregation may impact mental health may be unique in rural areas. The second analysis additionally adjusted for NDI to account for neighborhood-level characteristics. All analyses were conducted using SAS, version 9.4 (SAS Institute, Inc), and 2-sided *P* < .05 was considered statistically significant.

## Results

The final analytic sample included 201 115 pregnant individuals (mean [SD] age, 30.8 [5.3] years). In terms of race and ethnicity, 26.8% were Asian, 6.6% were Black, 28.0% were Hispanic, and 38.6% were White. Overall, Black individuals had the highest prevalence of depression (18.3%) and anxiety (18.4%), followed by White (16.0% for depression and 18.2% for anxiety), Hispanic (13.0% for depression and 14.4% for anxiety), and Asian (5.7% for depression and 6.4% for anxiety) individuals during pregnancy. Asian (40.8% vs 31.1%) and Black (43.3% vs 22.6%) individuals were more likely to live in neighborhoods with high (vs low) racial residential segregation, while Hispanic individuals were equally likely (34.3% vs 34.7%) to live in either. White individuals were more likely to live in neighborhoods with low (vs high) racial residential segregation (35.3% vs 25.9%) (Table). High compared with low racial residential segregation was associated with higher educational attainment (college degree or higher) among Asian (61.9% vs 58.5%) and White (50.7% vs 44.6%) individuals. High compared with low racial residential segregation was similarly associated with lower neighborhood deprivation among Asian (34.4% vs 33.9%) and White (54.3% vs 14.3%) individuals. In contrast, high compared with low racial residential segregation was associated with lower individual socioeconomic status, including educational attainment (college degree or higher) among Black (19.8% vs 32.3%) and Hispanic (15.5% vs 27.3%) individuals. Additionally, Medicaid was associated with high compared with low racial residential segregation (Black individuals, 35.4% vs 21.6%; Hispanic individuals, 17.3% vs 11.8%) as a proxy for income, as was higher neighborhood deprivation (NDI quartile 4) (Black individuals, 64.4% vs 18.2%; Hispanic individuals, 71.3% vs 15.6%) (Table).

**Table.** Characteristics of 201 115 Individuals With Singleton Pregnancies Resulting in Live Births at Kaiser Permanente Northern California, 2014 to 2019

Characteristic	Race and ethnicity by segregation category, No. (%)^a^											
	Asian (n = 53 914)			Black (n = 13 197)			Hispanic (n = 56 325)			White (n = 77 679)		
	Low	Medium	High	Low	Medium	High	Low	Medium	High	Low	Medium	High
All participants	16 786 (31.1)	15 120 (28.0)	22 008 (40.8)	2977 (22.6)	4500 (34.1)	5720 (43.3)	19 531 (34.7)	17 447 (31.0)	19 347 (34.3)	27 447 (35.3)	30 081 (38.7)	20 151 (25.9)
No. missing	<5	0	<5	0	0	0	0	0	0	0	0	0
**Sociodemographic information**												
Maternal age, mean (SD), y	32.5 (4.5)	32.1 (4.5)	32.0 (4.4)	30.3 (6.1)	29.2 (5.9)	28.7 (6.0)	29.8 (5.7)	29.1 (5.8)	28.6 (5.8)	30.9 (5.0)	31.3 (4.9)	31.6 (4.9)
Maternal educational level												
<12th Grade	164 (1.0)	191 (1.3)	277 (1.3)	54 (1.8)	108 (2.4)	153 (2.7)	772 (4.0)	1165 (6.7)	1617 (8.4)	332 (1.2)	182 (0.6)	75 (0.4)
High school graduate or GED	757 (4.5)	789 (5.2)	1135 (5.2)	295 (9.9)	604 (13.4)	927 (16.2)	2575 (13.2)	2970 (17.0)	3731 (19.3)	2193 (8.0)	1630 (5.4)	885 (4.4)
Some college or technical school	2303 (13.7)	2145 (14.2)	3173 (14.4)	852 (28.6)	1489 (33.1)	1835 (32.1)	5780 (29.6)	5362 (30.7)	5893 (30.5)	7370 (26.9)	6978 (23.2)	4227 (21.0)
College degree or higher	9827 (58.5)	8788 (58.1)	13 625 (61.9)	963 (32.3)	1124 (25.0)	1132 (19.8)	5325 (27.3)	3548 (20.3)	2995 (15.5)	12 234 (44.6)	14 661 (48.7)	10 220 (50.7)
Missing	3735 (22.3)	3207 (21.2)	3798 (17.3)	813 (27.3)	1175 (26.1)	1673 (29.2)	5079 (26.0)	4402 (25.2)	5111 (26.4)	5318 (19.4)	6630 (22.0)	4744 (23.5)
Medicaid insurance	953 (5.7)	794 (5.3)	1337 (6.1)	644 (21.6)	1334 (29.6)	2025 (35.4)	2306 (11.8)	2639 (15.1)	3345 (17.3)	2795 (10.2)	2162 (7.2)	1149 (5.7)
Partnered status												
Partnered	11 314 (67.4)	10 368 (68.6)	15 791 (71.8)	1193 (40.1)	1525 (33.9)	1513 (26.5)	9914 (50.8)	8441 (48.4)	9012 (46.6)	17 313 (63.1)	19 300 (64.2)	12 970 (64.4)
Not partnered	1461 (8.7)	1372 (9.1)	2132 (9.7)	888 (29.8)	1651 (36.7)	2319 (40.5)	3851 (19.7)	3865 (22.2)	4517 (23.3)	3934 (14.3)	3235 (10.8)	1837 (9.1)
Other	199 (1.2)	183 (1.2)	248 (1.1)	82 (2.8)	172 (3.8)	176 (3.1)	616 (3.2)	657 (3.8)	766 (4.0)	773 (2.8)	655 (2.2)	400 (2.0)
Unknown	3812 (22.7)	3197 (21.1)	3837 (17.4)	814 (27.3)	1152 (25.6)	1712 (29.9)	5150 (26.4)	4484 (25.7)	5052 (26.1)	5427 (19.8)	6891 (22.9)	4944 (24.5)
**Pregnancy health **												
Nulliparous	8283 (49.3)	7275 (48.1)	9878 (44.9)	1256 (42.2)	1815 (40.3)	2355 (41.2)	8039 (41.2)	6639 (38.1)	7142 (36.9)	12 845 (46.8)	13 748 (45.7)	9159 (45.5)
Smoking	165 (1.0)	138 (0.9)	195 (0.9)	103 (3.5)	140 (3.1)	216 (3.8)	243 (1.2)	195 (1.1)	192 (1.0)	898 (3.3)	732 (2.4)	454 (2.3)
Alcohol use	1443 (8.6)	1028 (6.8)	1389 (6.3)	226 (7.6)	317 (7.0)	474 (8.3)	1721 (8.8)	1401 (8.0)	1392 (7.2)	2679 (9.8)	2900 (9.6)	2001 (9.9)
Other substance use	190 (1.1)	158 (1.0)	230 (1.0)	86 (2.9)	138 (3.1)	195 (3.4)	329 (1.7)	307 (1.8)	358 (1.9)	587 (2.1)	554 (1.8)	353 (1.8)
**Neighborhood deprivation index quartile**												
1 (Lowest deprivation)	5694 (33.9)	5696 (37.7)	7571 (34.4)	736 (24.7)	376 (8.4)	188 (3.3)	5129 (26.3)	1396 (8.0)	189 (1.0)	3912 (14.3)	9737 (32.4)	10 935 (54.3)
2	4196 (25.0)	3825 (25.3)	6180 (28.1)	922 (31.0)	835 (18.6)	616 (10.8)	6104 (31.3)	3339 (19.1)	1062 (5.5)	6982 (25.4)	9857 (32.8)	5909 (29.3)
3	3492 (20.8)	2806 (18.6)	5186 (23.6)	778 (26.1)	1339 (29.8)	1234 (21.6)	5254 (26.9)	6147 (35.2)	4298 (22.2)	8739 (31.8)	7539 (25.1)	2516 (12.5)
4 (Highest deprivation)	3404 (20.3)	2793 (18.5)	3071 (14.0)	541 (18.2)	1950 (43.3)	3682 (64.4)	3044 (15.6)	6565 (37.6)	13 798 (71.3)	7814 (28.5)	2948 (9.8)	791 (3.9)

Abbreviation: GED, General Educational Development.

^a^ Data are stratified by race and ethnicity and neighborhood residential segregation category. Segregation category was determined using the Getis-Ord Gi* statistic.^26,27^ A statistic of greater than 1.96 indicates high segregation; 0 to 1.96, medium segregation; and less than 0, low segregation. Percentages have been rounded and may not total 100.

Among Asian individuals, living in a neighborhood with high (vs low) residential racial segregation was associated with lower odds of depression (adjusted odds ratio [AOR], 0.75 [95% CI, 0.69-0.82]) and anxiety (AOR, 0.80 [95% CI, 0.73-0.87]) during pregnancy (Figure and eTable 2 in Supplement 1). Similarly, among White individuals, living in a neighborhood with high (vs low) residential racial segregation was associated with lower odds of depression (AOR, 0.91 [95% CI, 0.86-0.96]), but not lower odds of anxiety (AOR, 0.95 [95% CI, 0.90-1.00]) during pregnancy. For Hispanic individuals, living in a neighborhood with high compared with low racial residential segregation was associated with lower odds of depression (AOR, 0.88 [95% CI, 0.82-0.94]) and anxiety (AOR, 0.88 [95% CI, 0.82-0.93]) during pregnancy. Among Black individuals, living in a neighborhood with high residential racial segregation was associated with higher odds of depression (AOR, 1.25 [95% CI, 1.10-1.42]) and anxiety (AOR, 1.14 [95% CI, 1.00-1.29]) (Figure and eTable 2 in Supplement 1).

**Figure.**
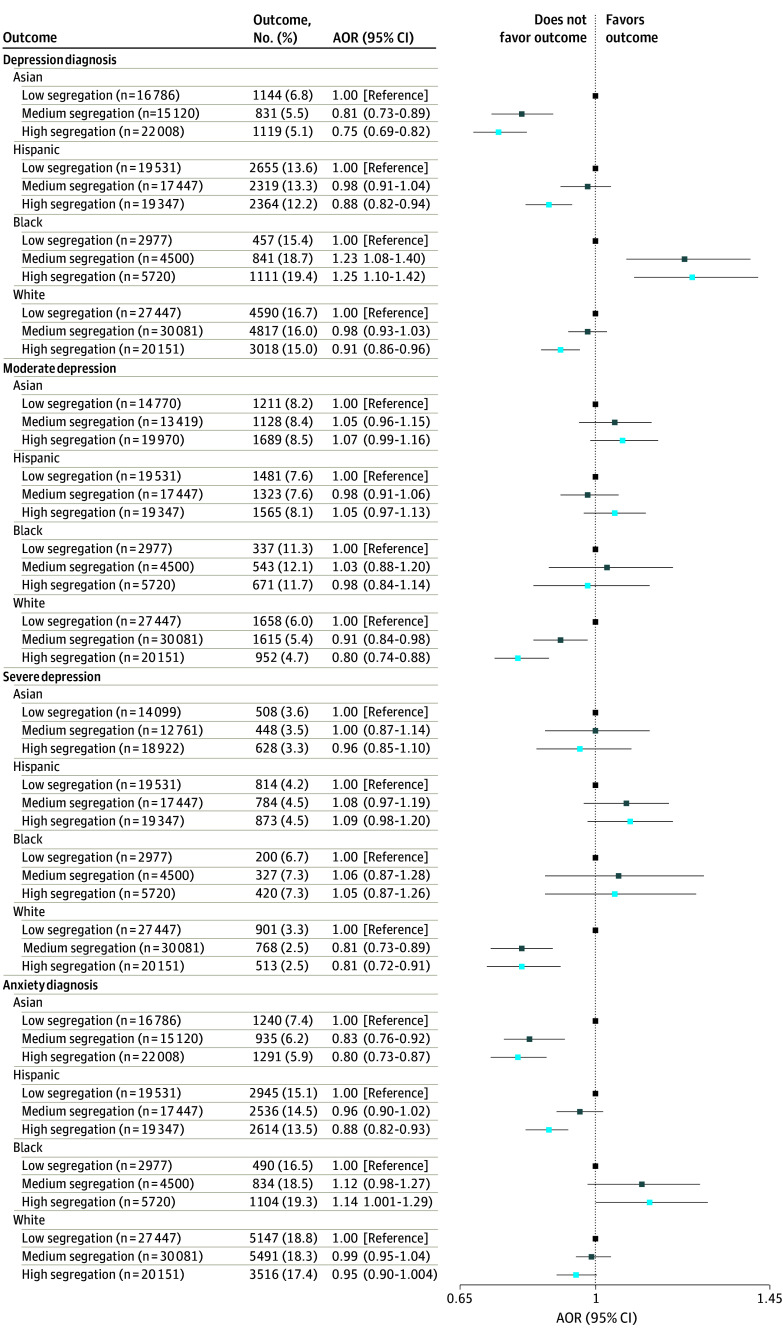
Association Between Racial Residential Segregation and Prenatal Mental Health Outcomes by Racial and Ethnic Group Adjusted odds ratios (AORs) are adjusted for maternal age, partnered status, smoking, alcohol use, and other substance use in early pregnancy.

High racial residential segregation was not associated with depression symptom severity among Asian, Hispanic, or Black individuals. Among White individuals, high racial residential segregation was associated with lower odds of moderate (AOR, 0.80 [95% CI, 0.74-0.88]) and severe (AOR, 0.81 [95% CI, 0.72-0.91]) depressive symptoms. Results from sensitivity analyses restricted to urban areas and additionally adjusting for NDI were similar to the main results (eTables 3 and 4 in Supplement 1).

## Discussion

In this large cross-sectional study of 201 115 pregnant individuals, we found that associations between racial residential segregation and prenatal mental health conditions differed by race and ethnicity. Among Black individuals, residing in highly segregated neighborhoods was associated with higher odds of prenatal depression and anxiety. In contrast for Asian and Hispanic individuals, high racial residential segregation was associated with lower odds of prenatal depression and anxiety. Residing in highly segregated neighborhoods was associated with lower odds of prenatal depression but not prenatal anxiety among White individuals. The different associations of racial residential segregation with prenatal mental health across racial and ethnic groups could be due to differing effects of racial residential segregation on economic and social factors in each group. While this study is among the first to explore this association, our findings support the growing body of literature demonstrating racial residential segregation is associated with health inequities between Black and White individuals.

The findings of a higher likelihood of prenatal depression and anxiety in Black pregnant individuals in our study echo those observed among nonpregnant Black adults,^38^ yet differ from a recent study conducted by Haight and colleagues in North Carolina,^23^ which suggested that pregnant Black individuals who resided in highly segregated neighborhoods have a lower risk of prenatal depression compared with pregnant Black individuals in less segregated neighborhoods. One explanation could be the differences in socioeconomic status between the 2 study populations, as Black pregnant individuals residing in highly segregated neighborhoods in the study by Haight and colleagues^23^ had a higher proportion of college graduates, while in our study Black individuals residing in neighborhoods of high (vs low) segregation had lower educational attainment and were more likely to have Medicaid insurance. Also, the former study was conducted in the Southern US, which historically has experienced heightened racial tension and more overt discrimination toward Black populations. Possibly, living among their same racial group in the South may limit the exposure to experiences of interpersonal racism for pregnant Black individuals in this area, creating a protective effect of segregation on mental health for Black populations. Our findings suggest that in Northern California, the negative economic effects of long-standing practices of institutional racism, such as racial residential segregation, could negate the protective cultural and social factors attributed to living among one’s race, especially for Black pregnant individuals.^22^

Contrary to pregnant Black individuals, high racial segregation for White individuals yielded protection against depression during pregnancy and no difference for anxiety. Institutional policies, such as redlining, created a multigenerational beneficial effect for residential areas of higher White populations, deeming them more suitable areas for economic investment and sustainability.^39,40^ Thus, these areas often have lower neighborhood deprivation (as observed in this study) and greater accessibility to health care services.^41^ Individuals living in these areas are less likely to encounter stressful life events (ie, financial stress, crime)^40^ that may exacerbate the likelihood of mental health conditions, especially during pregnancy.

For pregnant Asian individuals, high racial residential segregation was associated with lower odds of both prenatal depression and anxiety. Previous research has similarly documented that for Asian adults, living among a higher concentration of one’s ethnic group was protective against depression and anxiety.^20^ One explanation for this protective effect could be the capacity of Asian populations to build economic capital that can be used to create favorable neighborhood qualities and enhance positive social relations, as observed in prior research.^42^ In this study, less neighborhood deprivation was observed among high (vs low) segregated neighborhoods among Asian individuals. Possibly, among Asian populations, living in high racial residential segregated areas provides an opportunity for affordable housing and increased social ties and economic investment while maintaining cultural norms and identities, thus reducing their vulnerability to psychological distress, discrimination, and experience of mental health conditions.

Pregnant Hispanic individuals residing in areas of high racial residential segregation (also more deprived) had lower odds of depression and anxiety during pregnancy. The ethnic density hypothesis may explain this association, as living among higher concentrations of one’s ethnic group may curate strong social support, reduce negative experiences (eg, discrimination), and buffer against negative economic impacts, thus reducing mental health conditions among Hispanic pregnant individuals. Additionally, our findings may also be attributed to the documented differences in risk of prenatal mental health conditions by racial and ethnic subgroups and birthplaces within the Hispanic group.^43,44^

Research on the intersectionality among race and ethnicity, nativity, and mental health is needed to further understand the associations documented in our study. Compared with Asian and Hispanic populations, Black US residents may experience more detrimental impacts from segregated neighborhoods. However, factors related to nativity (eg, US-born vs non–US-born status, immigrant status, and acculturation) may contribute to the association between racial residential segregation and lower risk of prenatal mental health conditions among Asian and Hispanic individuals observed in this study. Previous studies have documented lower risk for poor mental health in non–US-born Hispanic individuals.^44^

### Strengths and Limitations

This study has several strengths, including a large, diverse population covering a 22-county region, with race and ethnicity defined by self-report. Additionally, KPNC’s universal perinatal mental health screening program^45^ uniquely documents depression symptom severity and provides a population screened for mental health diagnosis, thereby reducing the potential for recall and misclassification bias. We used a widely accepted measure of racial residential segregation in health outcomes research,^29,30,31,32,33,34^ the Gi* statistic.^24^

This study also had limitations. Participants were limited to pregnant individuals receiving care through KPNC, an insured population; therefore, the findings may not be generalizable to uninsured populations. The study did not account for maternal nativity status (US born vs non–US born), immigrant status (eg, refugee or asylee), or subgroups of the Hispanic (eg, Mexican) or Asian (eg, Chinese) populations. This study did not measure sociocultural factors (such as social cohesion) that may differentiate the effects of racial residential segregation on mental health conditions within racial and ethnic groups. Further, this study does not account for other important factors such as length of residency in a particular neighborhood prior to and or during pregnancy, or whether depression and anxiety diagnoses existed prior to pregnancy and thus persisted. Thus, this study cannot establish causality between racial residential segregation and prenatal mental health conditions. Last, we excluded American Indian or Alaska Native and Native Hawaiian or Other Pacific Islander groups due to the small sample sizes; we excluded multiracial pregnant individuals given the heterogeneity within this group. Future research is needed to explore the complex association between structural factors and mental health in those with multiracial identity. Future studies should explore how these associations differ by subgroups and birthplace of racial and ethnic groups. Further, the role of individual (eg, income) and neighborhood sociocultural and socioeconomic (eg, social cohesion and neighborhood deprivation) characteristics should be investigated as potential mediators in the causal pathway between racial residential segregation and prenatal mental health conditions.

## Conclusions

This cross-sectional study provides evidence of an association between residential segregation and prenatal mental health conditions that differs by racial and ethnic groups, suggesting worse prenatal mental health for Black individuals and better mental health for Asian, Hispanic, and White individuals. These findings emphasize the need for research to explore the intersectional mechanisms underlying these associations, with special attention to the disparity between Black and White individuals. Implementation of social and public health policies that prioritize investment in neighborhoods with high segregation may improve prenatal mental health outcomes and reduce ongoing health disparities in the US, particularly among pregnant Black individuals.

## Notes

Supplement 1. **eTable 1.** *International Classification of Diseases, Ninth Revision, Clinical Modification* (*ICD-9*) and *International Statistical Classification of Diseases, Tenth Revision, Clinical Modification* (*ICD-10*) Codes for Depression and Anxiety **eTable 2.** Associations Between Racial Residential Segregation and Prenatal Mental Health Outcomes by Racial and Ethnic Group (Full Model Estimates) **eTable 3.** Associations of Racial Residential Segregation and Prenatal Mental Health Outcomes by Racial and Ethnic Groups in Urban Areas **eTable 4.** Associations Between Racial Residential Segregation and Prenatal Mental Health Outcomes by Racial and Ethnic Group (Full Model Estimates Additionally Adjusted for NDI) **eFigure.** Association Between Racial Residential Segregation and Prenatal Mental Health Outcomes by Racial and Ethnic Group (Adjusted for NDI)

Supplement 2.
Data Sharing Statement
