# Supplementary material for: Racial Residential Segregation and Mental Health During Pregnancy
Source: JAMA Health Forum. 2024 Oct 25;5(10):e243669. doi: 10.1001/jamahealthforum.2024.3669 (PMC11581653; doi:10.1001/jamahealthforum.2024.3669)
Supplement: Supplement 1. — eTable 1. International Classification of Diseases, Ninth Revision, Clinical Modification (ICD-9) and International Statistical Classification of Diseases, Tenth Revision, Clinical Modification (ICD-10) Codes for Depression and Anxiety eTable 2. Associations Between Racial Residential Segregation and Prenatal Mental Health Outcomes by Racial and Ethnic Group (Full Model Estimates) eTable 3. Associations of Racial Residential Segregation and Prenatal Mental Health Outcomes by Racial and Ethnic Groups in Urban Areas eTable 4. Associations Between Racial Residential Segregation and Prenatal Mental Health Outcomes by Racial and Ethnic Group (Full Model Estimates Additionally Adjusted for NDI) eFigure. Association Between Racial Residential Segregation and Prenatal Mental Health Outcomes by Racial and Ethnic Group (Adjusted for NDI) [file jamahealthforum-e243669-s001.pdf]

Supplementary Online Content

Kelly-Taylor K, Badon SE, Dyer WT, et al. Racial residential segregation and mental health during pregnancy. *JAMA Health Forum*. Published October 25, 2024. doi:10.1001/jamahealthforum.2024.3669

eTable 1. *International Classification of Diseases, Ninth Revision, Clinical Modification* (*ICD-9*) and *International Statistical Classification of Diseases, Tenth Revision, Clinical Modification* (*ICD-10*) Codes for Depression and Anxiety

eTable 2. Associations Between Racial Residential Segregation and Prenatal Mental Health Outcomes by Racial and Ethnic Group (Full Model Estimates)

eTable 3. Associations of Racial Residential Segregation and Prenatal Mental Health Outcomes by Racial and Ethnic Groups in Urban Areas

eTable 4. Associations Between Racial Residential Segregation and Prenatal Mental Health Outcomes by Racial and Ethnic Group (Full Model Estimates Additionally Adjusted for NDI)

eFigure. Association Between Racial Residential Segregation and Prenatal Mental Health Outcomes by Racial and Ethnic Group (Adjusted for NDI)

This supplementary material has been provided by the authors to give readers additional information about their work.

© 2024 Kelly-Taylor K et al. *JAMA Health Forum.*

eTable 1. *International Classification of Diseases, Ninth Revision, Clinical Modification* (*ICD-9*) and *International Statistical Classification of Diseases, Tenth Revision, Clinical Modification* (*ICD-10*) Codes for Depression and Anxiety

	**Depression**	**Anxiety**
**ICD-9**	296.2, 296.21, 296.22, 296.23, 296.24, 296.25, 296.3, 296.31, 296.32, 296.33, 296.34, 296.35, 296.82, 298, 300.4, 309.1, 309.28, 311, 648.4, 648.41, 648.42, 648.43, 648.44	293.84, 300.09, 309.24, 309.28, 648.4, 648.41, 648.42, 648.43, 648.44
**ICD-10**	F32.0, F32.1, F32.2, F32.3, F32.4, F32.8, F32.81, F32.89, F32.9, F33.0, F33.1, F33.2, F33.3, F33.41, F33.8, F33.9, F34.1, F43.21, F43.23, F53.0, O99.34, O99.340, O99.341, O99.342, O99.343, O99.344, O99.345, O90.6	F06.4, F41.8, F41.9, F43.22, F43.23, O99.34, O99.340, O99.341, O99.342, O99.343, O99.344, O99.345

© 2024 Kelly-Taylor K et al. *JAMA Health Forum.*

eTable 2. Associations Between Racial Residential Segregation and Prenatal Mental Health Outcomes by Racial and Ethnic Group (Full Model Estimates)

	OR (95% CI)			
	Asian (n=53,914)	Hispanic(n=56,325)	Non-Hispanic Black(n=13,197)	Non-Hispanic white(n=77,679)
Outcome: Depression				
Residential segregation status				
Low residential segregation	Referent	Referent	Referent	Referent
Medium residential segregation	0.81(0.73-0.89)	0.98(0.91-1.04)	1.23(1.08-1.40)	0.98(0.93-1.03)
High residential segregation	0.75(0.69-0.82)	0.88(0.82-0.94)	1.25(1.10-1.42)	0.91(0.86-0.96)
Maternal age	1.02(1.01-1.03)	1.01(1.01-1.02)	0.99(0.98-1.00)	1.00(1.00-1.01)
Partnered status				
Partnered	Referent	Referent	Referent	Referent
Unpartnered	1.69(1.51-1.90)	1.60(1.50-1.71)	1.45(1.29-1.63)	1.77(1.67-1.88)
Other/unknown partnered status	1.21(1.10-1.32)	1.28(1.20-1.36)	1.19(1.05-1.34)	1.16(1.11-1.22)
Alcohol use (yes vs no)	1.26(1.11-1.44)	1.18(1.08-1.29)	1.20(1.02-1.42)	1.06(0.99-1.13)
Smoking status (yes vs no)	2.28(1.75-2.98)	2.52(2.10-3.02)	2.00(1.61-2.48)	2.14(1.93-2.36)
Other substance use (yes vs no)	2.15(1.66-2.78)	2.40(2.07-2.78)	1.79(1.43-2.24)	2.41(2.14-2.70)
Outcome: Moderate depression severity				
Residential segregation status				
Low residential segregation	Referent	Referent	Referent	Referent
Medium residential segregation	1.05(0.96-1.15)	0.98(0.91-1.06)	1.03(0.88-1.20)	0.91(0.84-0.98)
High residential segregation	1.07(0.99-1.16)	1.05(0.97-1.13)	0.98(0.84-1.14)	0.80(0.74-0.88)
Maternal age	0.98(0.97-0.99)	0.99(0.99-1.00)	0.98(0.97-0.99)	0.98(0.97-0.98)
Partnered status				
Partnered	Referent	Referent	Referent	Referent
Unpartnered	1.38(1.24-1.53)	1.47(1.36-1.60)	1.56(1.34-1.80)	1.58(1.44-1.73)
Other/Unknown partnered status	1.11(1.02-1.21)	1.21(1.13-1.31)	1.47(1.27-1.71)	1.26(1.17-1.36)
Alcohol use (yes vs no)	1.05(0.92-1.19)	1.27(1.14-1.41)	1.22(1.00-1.48)	1.21(1.10-1.34)
Smoking status (yes vs no)	1.91(1.46-2.50)	1.93(1.53-2.43)	2.08(1.61-2.69)	1.94(1.68-2.25)
Other substance use (yes vs no)	1.68(1.29-2.18)	1.90(1.57-2.29)	1.69(1.29-2.22)	2.17(1.84-2.58)
Outcome: Severe depression severity				
Residential segregation status				
Low residential segregation	Referent	Referent	Referent	Referent
Medium residential segregation	1.00(0.87-1.15)	1.08(0.97-1.19)	1.06(0.87-1.28)	0.81(0.73-0.89)
High residential segregation	0.96(0.84-1.09)	1.09(0.98-1.20)	1.05(0.87-1.26)	0.81(0.72-0.91)
Maternal age	0.99(0.98-1.00)	1.01(1.01-1.02)	1.01(1.00-1.02)	0.98(0.97-0.99)
Partnered status				

© 2024 Kelly-Taylor K et al. *JAMA Health Forum.*

	OR (95% CI)			
	Asian (n=53,914)	Hispanic(n=56,325)	Non-Hispanic Black(n=13,197)	Non-Hispanic white(n=77,679)
Partnered	Referent	Referent	Referent	Referent
Unpartnered	1.80(1.54-2.11)	1.75(1.57-1.95)	1.94(1.62-2.32)	2.15(1.91-2.42)
Other/unknown partnered status	1.41(1.24-1.59)	1.59(1.44-1.75)	1.36(1.13-1.65)	1.52(1.37-1.68)
Alcohol use (yes vs no)	0.95(0.78-1.17)	1.28(1.11-1.47)	1.11(0.87-1.41)	1.03(0.90-1.19)
Smoking status (yes vs no)	1.82(1.22-2.74)	2.54(1.94-3.32)	2.58(1.93-3.45)	2.98(2.52-3.52)
Other substance use (yes vs no)	1.91(1.30-2.80)	2.00(1.58-2.54)	1.57(1.12-2.20)	3.29(2.72-3.98)
Outcome: Anxiety				
Residential segregation status				
Low residential segregation	Referent	Referent	Referent	Referent
Medium residential segregation	0.84(0.76-0.92)	0.96(0.90-1.02)	1.12(0.98-1.27)	0.99(0.95-1.04)
High residential segregation	0.80(0.73-0.87)	0.88(0.82-0.93)	1.14(1.00-1.29)	0.95(0.90-1.00)
Maternal age	1.02(1.01-1.03)	1.01(1.01-1.01)	0.99(0.99-1.00)	1.00(1.00-1.01)
Partnered status				
Partnered	Referent	Referent	Referent	Referent
Unpartnered	1.61(1.44-1.79)	1.49(1.39-1.58)	1.49(1.33-1.68)	1.73(1.63-1.83)
Other/unknown partnered status	1.17(1.08-1.28)	1.20(1.13-1.27)	1.22(1.08-1.38)	1.17(1.11-1.22)
Alcohol use (yes vs no)	1.27(1.12-1.44)	1.21(1.11-1.32)	1.15(0.97-1.35)	1.04(0.98-1.11)
Smoking status (yes vs no)	2.29(1.77-2.95)	2.34(1.96-2.81)	1.92(1.55-2.38)	2.13(1.93-2.35)
Other substance use (yes vs no)	2.11(1.65-2.71)	2.35(2.03-2.72)	1.83(1.46-2.29)	2.46(2.20-2.76)

© 2024 Kelly-Taylor K et al. *JAMA Health Forum.*

eTable 3. Associations of Racial Residential Segregation and Prenatal Mental Health Outcomes by Racial and Ethnic Groups in Urban Areas

	OR[1] (95% CI)			
	**Asian** (n=53,914)	**Hispanic** (n=54,408)	**Non-Hispanic Black** (n=13,130)	**Non-Hispanic white** (n=75,713)
Outcome: Depression diagnosis				
Low residential segregation	Referent	Referent	Referent	Referent
Medium residential segregation	0.81 (0.65-1.01)	1.00 (0.93-1.07)	1.24 (1.08-1.41)	0.97 (0.93-1.02)
High residential segregation	0.68 (0.55-0.84)	0.88 (0.82-0.94)	1.25 (1.10-1.43)	0.92 (0.87-0.97)
Outcome: Moderate depression severity[2]				
Low residential segregation	Referent	Referent	Referent	Referent
Medium residential segregation	1.09 (0.90-1.32)	0.98 (0.90-1.06)	1.02 (0.88-1.19)	0.92 (0.85-0.99)
High residential segregation	1.03 (0.86-1.23)	1.06 (0.98-1.14)	0.98 (0.84-1.14)	0.80 (0.73-0.88)
Outcome: Severe depression severity[2]				
Low residential segregation	Referent	Referent	Referent	Referent
Medium residential segregation	1.00 (0.87-1.16)	1.08 (0.97-1.20)	1.05 (0.87-1.27)	0.81 (0.73-0.90)
High residential segregation	0.96 (0.84-1.10)	1.09 (0.99-1.21)	1.04 (0.86-1.25)	0.80 (0.71-0.90)
Outcome: Anxiety diagnosis				
Low residential segregation	Referent	Referent	Referent	Referent
Medium residential segregation	0.84 (0.76-0.92)	0.97 (0.91-1.04)	1.12 (0.98-1.28)	0.99 (0.94-1.04)
High residential segregation	0.80 (0.73-0.87)	0.88 (0.82-0.94)	1.14 (1.001-1.29)	0.96 (0.91-1.01)

[1]Adjusted for maternal age, partnered status, smoking, alcohol use, and other substance use during pregnancy
[2]Mild depression severity is the reference group

© 2024 Kelly-Taylor K et al. *JAMA Health Forum.*

eTable 4. Associations Between Racial Residential Segregation and Prenatal Mental Health Outcomes by Racial and Ethnic Group (Full Model Estimates Additionally Adjusted for NDI)

	OR (95% CI)			
	Asian (n=53,914)	Hispanic(n=56,325)	Non-Hispanic Black(n=13,197)	Non-Hispanic white(n=77,679)
**Outcome: Depression**				
Residential segregation status				
Low residential segregation	Referent	Referent	Referent	Referent
Medium residential segregation	0.81(0.73-0.89)	1.02(0.95-1.10)	1.18(1.03-1.35)	0.97(0.92-1.02)
High residential segregation	0.74(0.68-0.82)	0.96(0.89-1.05)	1.18(1.02-1.35)	0.91(0.85-0.96)
Maternal age	1.02(1.01-1.03)	1.01(1.01-1.02)	0.99(0.98-1.00)	1.00(1.00-1.01)
Partnered status				
Partnered	Referent	Referent	Referent	Referent
Unpartnered	1.72(1.53-1.93)	1.61(1.51-1.72)	1.44(1.28-1.62)	1.77(1.67-1.88)
Other/unknown partnered status	1.22(1.11-1.33)	1.29(1.21-1.37)	1.18(1.05-1.34)	1.16(1.11-1.22)
Alcohol use (yes vs no)	1.25(1.10-1.43)	1.17(1.07-1.28)	1.21(1.03-1.43)	1.06(0.99-1.13)
Smoking status (yes vs no)	2.29(1.76-2.99)	2.52(2.10-3.02)	1.98(1.60-2.46)	2.14(1.93-2.37)
Other substance use (yes vs no)	2.15(1.66-2.78)	2.41(2.08-2.80)	1.78(1.42-2.23)	2.41(2.14-2.70)
Neighborhood deprivation				
Quartile 1 (least deprived)	Referent	Referent	Referent	Referent
Quartile 2	1.13(1.02-1.25)	0.95(0.87-1.05)	1.20(0.99-1.47)	1.01(0.95-1.07)
Quartile 3	1.03(0.92-1.14)	0.91(0.83-1.00)	1.25(1.03-1.51)	0.99(0.93-1.05)
Quartile 4	0.93(0.82-1.04)	0.82(0.74-0.90)	1.28(1.06-1.54)	0.99(0.92-1.06)
**Outcome: Moderate depression severity**				
Residential segregation status				
Low residential segregation	Referent	Referent	Referent	Referent
Medium residential segregation	1.05(0.96-1.15)	0.95(0.87-1.03)	0.96(0.82-1.12)	0.96(0.89-1.04)
High residential segregation	1.08(0.99-1.17)	0.98(0.89-1.07)	0.89(0.76-1.05)	0.87(0.79-0.96)
Maternal age	0.98(0.98-0.99)	0.99(0.99-1.00)	0.98(0.97-0.99)	0.98(0.97-0.99)
Partnered status				
Partnered	Referent	Referent	Referent	Referent
Unpartnered	1.35(1.21-1.51)	1.47(1.36-1.60)	1.54(1.33-1.78)	1.56(1.42-1.71)
Other/unknown partnered status	1.11(1.02-1.20)	1.21(1.12-1.30)	1.46(1.26-1.69)	1.24(1.15-1.34)
Alcohol use (yes vs no)	1.05(0.93-1.19)	1.28(1.15-1.42)	1.23(1.01-1.49)	1.23(1.11-1.36)
Smoking status (yes vs no)	1.99(1.45-2.48)	1.92(1.52-2.43)	2.05(1.59-2.66)	1.91(1.65-2.22)
Other substance use (yes vs no)	1.68(1.29-2.18)	1.89(1.57-2.29)	1.68(1.28-2.20)	2.15(1.82-2.55)
Neighborhood deprivation				
Quartile 1 (least deprived)	Referent	Referent	Referent	Referent

© 2024 Kelly-Taylor K et al. *JAMA Health Forum.*

	OR (95% CI)			
	Asian (n=53,914)	Hispanic(n=56,325)	Non-Hispanic Black(n=13,197)	Non-Hispanic white(n=77,679)
Quartile 2	1.03(0.95-1.13)	0.99(0.87-1.12)	1.33(1.04-1.70)	1.05(0.96-1.14)
Quartile 3	1.06(0.97-1.17)	1.12(0.99-1.26)	1.44(1.13-1.82)	1.06(0.97-1.17)
Quartile 4	1.11(1.002-1.23)	1.14(1.01-1.29)	1.48(1.17-1.86)	1.32(1.19-1.47)
**Outcome: Severe depression severity**				
Residential segregation status				
Low residential segregation	Referent	Referent	Referent	Referent
Medium residential segregation	1.01(0.88-1.16)	1.02(0.91-1.13)	0.98(0.81-1.20)	0.87(0.78-0.97)
High residential segregation	0.97(0.85-1.10)	1.00(0.89-1.12)	0.96(0.78-1.17)	0.92(0.81-1.04)
Maternal age	0.99(0.98-1.001)	1.01(1.01-1.02)	1.01(1.00-1.02)	0.98(0.97-0.99)
Partnered status				
Partnered	Referent	Referent	Referent	Referent
Unpartnered	1.71(1.45-2.00)	1.74(1.56-1.94)	1.92(1.60-2.29)	2.11(1.87-2.37)
Other/unknown partnered status	1.38(1.22-1.57)	1.57(1.43-1.74)	1.36(1.12-1.64)	1.48(1.34-1.64)
Alcohol use (yes vs no)	0.97(0.79-1.19)	1.29(1.13-1.48)	1.13(0.89-1.44)	1.05(0.91-1.21)
Smoking status (yes vs no)	1.79(1.19-2.69)	2.53(1.94-3.31)	2.54(1.90-3.40)	2.92(2.48-3.45)
Other substance use (yes vs no)	1.89(1.29-2.78)	2.00(1.58-2.53)	1.55(1.11-2.17)	3.25(2.69-3.93)
Neighborhood deprivation				
Quartile 1 (least deprived)	Referent	Referent	Referent	Referent
Quartile 2	1.10(0.95-1.27)	1.23(1.04-1.45)	1.34(0.99-1.82)	1.04(0.92-1.18)
Quartile 3	1.21(1.04-1.41)	1.35(1.15-1.58)	1.56(1.16-2.10)	1.15(1.01-1.31)
Quartile 4	1.37(1.17-1.60)	1.34(1.13-1.57)	1.49(1.12-2.00)	1.45(1.25-1.67)
**Outcome: Anxiety**				
Residential segregation status				
Low residential segregation	Referent	Referent	Referent	Referent
Medium residential segregation	0.84(0.76-0.92)	1.01(0.94-1.08)	1.06(0.92-1.22)	0.99(0.94-1.04)
High residential segregation	0.79(0.73-0.87)	0.98(0.91-1.06)	1.06(0.92-1.21)	0.95(0.89-1.01)
Maternal age	1.02(1.01-1.02)	1.01(1.00-1.01)	1.00(0.99-1.00)	1.00(1.00-1.01)
Partnered status				
Partnered	Referent	Referent	Referent	Referent
Unpartnered	1.63(1.45-1.81)	1.49(1.40-1.59)	1.48(1.31-1.67)	1.73(1.63-1.83)
Other/unknown partnered status	1.18(1.08-1.28)	1.21(1.14-1.28)	1.22(1.08-1.38)	1.17(1.11-1.22)
Alcohol use (yes vs no)	1.26(1.12-1.43)	1.20(1.10-1.31)	1.16(0.98-1.37)	1.04(0.98-1.11)
Smoking status (yes vs no)	2.29(1.78-2.96)	2.35(1.96-2.81)	1.90(1.53-2.35)	2.13(1.93-2.35)
Other substance use (yes vs no)	2.11(1.65-2.71)	2.36(2.04-2.73)	1.81(1.45-2.27)	2.46(2.20-2.76)
Neighborhood deprivation				

© 2024 Kelly-Taylor K et al. *JAMA Health Forum.*

	OR (95% CI)			
	Asian (n=53,914)	Hispanic(n=56,325)	Non-Hispanic Black(n=13,197)	Non-Hispanic white(n=77,679)
Quartile 1 (least deprived)	Referent	Referent	Referent	Referent
Quartile 2	1.14(1.04-1.25)	0.94(0.86-1.03)	1.22(1.00-1.49)	1.01(0.95-1.06)
Quartile 3	1.04(0.94-1.15)	0.91(0.83-0.99)	1.34(1.11-1.63)	0.98(0.92-1.04)
Quartile 4	0.95(0.85-1.06)	0.78(0.71-0.86)	1.33(1.10-1.61)	1.01(0.94-1.08)

© 2024 Kelly-Taylor K et al. *JAMA Health Forum.*

eFigure 1. Association Between Racial Residential Segregation and Prenatal Mental Health Outcomes by Racial and Ethnic Group (Adjusted for NDI)*

Group	Outcome n (%)	aOR**	95% CI
**Outcome: Depression diagnosis**			
Asian			
Low segregation (n=16,786)	1144 (6.8)	1.00	1.00 [reference]
Medium segregation (n=15,120)	831 (5.5)	0.81	0.74, 0.89
High segregation (n=22,008)	1119 (5.1)	0.74	0.68, 0.82
Hispanic			
Low segregation (n=19,531)	2655 (34.7)	1.00	1.00 [reference]
Medium segregation (n=17,447)	2319 (13.3)	1.02	0.95, 1.10
High segregation (n=19,347)	2364 (12.2)	0.96	0.89, 1.05
Black			
Low segregation (n=2977)	457 (15.4)	1.00	1.00 [reference]
Medium segregation (n=4500)	841 (18.7)	1.18	1.03, 1.35
High segregation (n=5720)	1111 (19.4)	1.18	1.02, 1.35
White			
Low segregation (n=27,447)	4590 (16.7)	1.00	1.00 [reference]
Medium segregation (n=30,081)	4817 (16.0)	0.97	0.92, 1.02
High segregation (n=20,151)	3018 (15.0)	0.91	0.85, 0.96
**Outcome: Moderate depression severity**			
Asian			
Low segregation (n=14,770)	1211 (6.9)	1.00	1.00 [reference]
Medium segregation (n=13,419)	1128 (7.2)	1.05	0.96, 1.15
High segregation (n=19,970)	1689 (7.5)	1.08	0.99, 1.17
Hispanic			
Low segregation (n=19,531)	1481 (7.6)	1.00	1.00 [reference]
Medium segregation (n=17,447)	1323 (7.6)	0.95	0.87, 1.03
High segregation (n=19,347)	1565 (8.1)	0.98	0.89, 1.07
Black			
Low segregation (n=2486)	337 (11.3)	1.00	1.00 [reference]
Medium segregation (n=3760)	543 (12.1)	0.96	0.82, 1.12
High segregation (n=4645)	671 (11.7)	0.89	0.76, 1.05
White			
Low segregation (n=24,386	1658 (6.0)	1.00	1.00 [reference]
Medium segregation (n=26,765)	1615 (5.4)	0.96	0.89, 1.04
High segregation (n=17,989)	952 (4.7)	0.87	0.79, 0.96
**Outcome: Severe depression severity**			
Asian			
Low segregation (n=14,099)	508 (2.9)	1.00	1.00 [reference]
Medium segregation (n=12,761	448 (2.9)	1.01	0.88, 1.16
High segregation (n=18,922)	628 (2.8)	0.97	0.85, 1.10
Hispanic			
Low segregation (n=16,458)	814 (4.2)	1.00	1.00 [reference]
Medium segregation (n=14,751)	784 (4.5)	1.02	0.91, 1.13
High segregation (n=16,292)	873 (4.5)	1.00	0.89, 1.12
Black			
Low segregation (n=2349)	200 (6.7)	1.00	1.00 [reference]
Medium segregation (n=3544)	327 (7.3)	0.98	0.81, 1.20
High segregation (n=4394)	420 (7.3)	0.96	0.78, 1.17
White			
Low segregation (n=23,629	901 (3.3)	1.00	1.00 [reference]
Medium segregation (n=25,918)	768 (2.6)	0.87	0.78, 0.97
High segregation (n=17,550)	513 (2.5)	0.92	0.81, 1.04

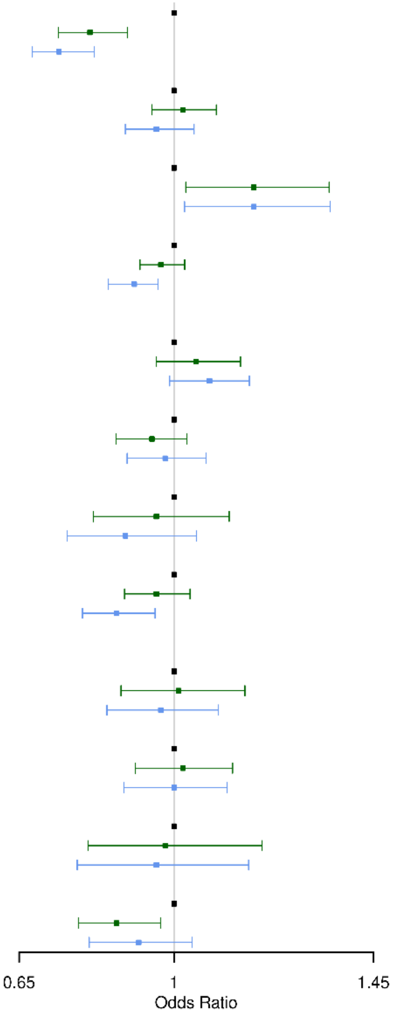

Adjusted for maternal age, partnered status, smoking, alcohol use, neighborhood deprivation, and other substance use in early pregnancy

© 2024 Kelly-Taylor K et al. *JAMA Health Forum.*

eFigure 1, cont. Association Between Racial Residential Segregation and Prenatal Mental Health Outcomes by Racial and Ethnic Group (Adjusted for NDI)

Group	Outcome n (%)	aOR**	95% CI
**Outcome: Anxiety diagnosis**			
Asian			
Low segregation (n=16,786)	1240 (7.4)	1.00	1.00 [reference]
Medium segregation (n=15,120)	935 (6.2)	0.84	0.76, 0.92
High segregation (n=22,008)	1291 (5.9)	0.79	0.73, 0.87
Hispanic			
Low segregation (n=19,531)	2945 (15.1)	1.00	1.00 [reference]
Medium segregation (n=17,447)	2536 (14.5)	1.01	0.94, 1.08
High segregation (n=19,347)	2614 (13.5)	0.98	0.91, 1.06
Black			
Low segregation (n=2977)	490 (16.5)	1.00	1.00 [reference]
Medium segregation (n=4500)	834 (18.5)	1.06	0.92, 1.22
High segregation (n=5720)	1104 (19.3)	1.06	0.92, 1.21
White			
Low segregation (n=27,447)	5147 (18.8)	1.00	1.00 [reference]
Medium segregation (n=30,081)	5491 (18.3)	0.99	0.95, 1.04
High segregation (n=20,151)	3516 (17.4)	0.95	0.89, 1.01

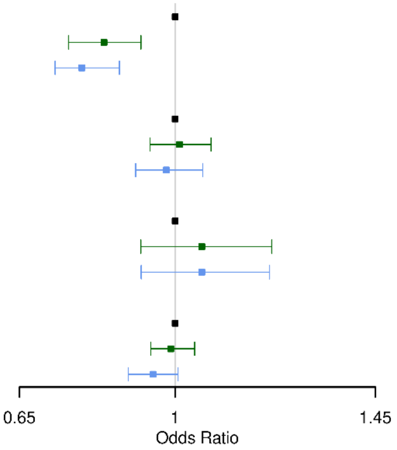

**Adjusted for maternal age, partnered status, smoking, alcohol use, neighborhood deprivation, and other substance use in early pregnancy*

© 2024 Kelly-Taylor K et al. *JAMA Health Forum.*
