# Supplementary material for: Racial Residential Segregation and Mental Health During Pregnancy
Source: JAMA Health Forum. 2024 Oct 25;5(10):e243669. doi: 10.1001/jamahealthforum.2024.3669 (PMC11581653; doi:10.1001/jamahealthforum.2024.3669)
Supplement: Supplement 2. — Data Sharing Statement [file jamahealthforum-e243669-s002.pdf]

Data Sharing Statement

Kelly-Taylor. Racial Residential Segregation and Mental Health During Pregnancy. *JAMA Health Forum*. Published October 25, 2024. doi:10.1001/jamahealthforum.2024.3669

Data
Data available: No
